# Implications of Sensory Processing and Attentional Differences Associated With Autism in Academic Settings: An Integrative Review

**DOI:** 10.3389/fpsyt.2021.695825

**Published:** 2021-08-27

**Authors:** Courtney Mallory, Brandon Keehn

**Affiliations:** ^1^Department of Speech, Language, and Hearing Sciences, Purdue University, West Lafayette, IN, United States; ^2^Department of Psychological Sciences, Purdue University, West Lafayette, IN, United States

**Keywords:** autism spectrum disorder, attention, education, academic settings, sensory processing abnormalities, academics

## Abstract

The impact of classroom environments on student engagement and academic performance is well-documented. Autism spectrum disorder (ASD) is associated with atypical sensory processing and attentional impairments, which may lead to challenges in successfully accessing educational material within these settings. These symptoms may help explain why students with ASD show discrepancies between intellectual ability and academic performance. Given the increasing number of students with ASD present in classrooms, understanding strengths and weaknesses in sensory processing and attention is necessary in order to design better classroom environments and develop more efficacious accommodations and interventions to support optimal student success. Therefore, the objectives of this review are to provide a brief review of the current literature on sensory processing and attention in ASD, survey how sensory and attentional functions affect academic outcomes in both neurotypical and ASD learners, and suggest potential accommodations/interventions for students with ASD based on these findings.

## Introduction

**Figure 1:**
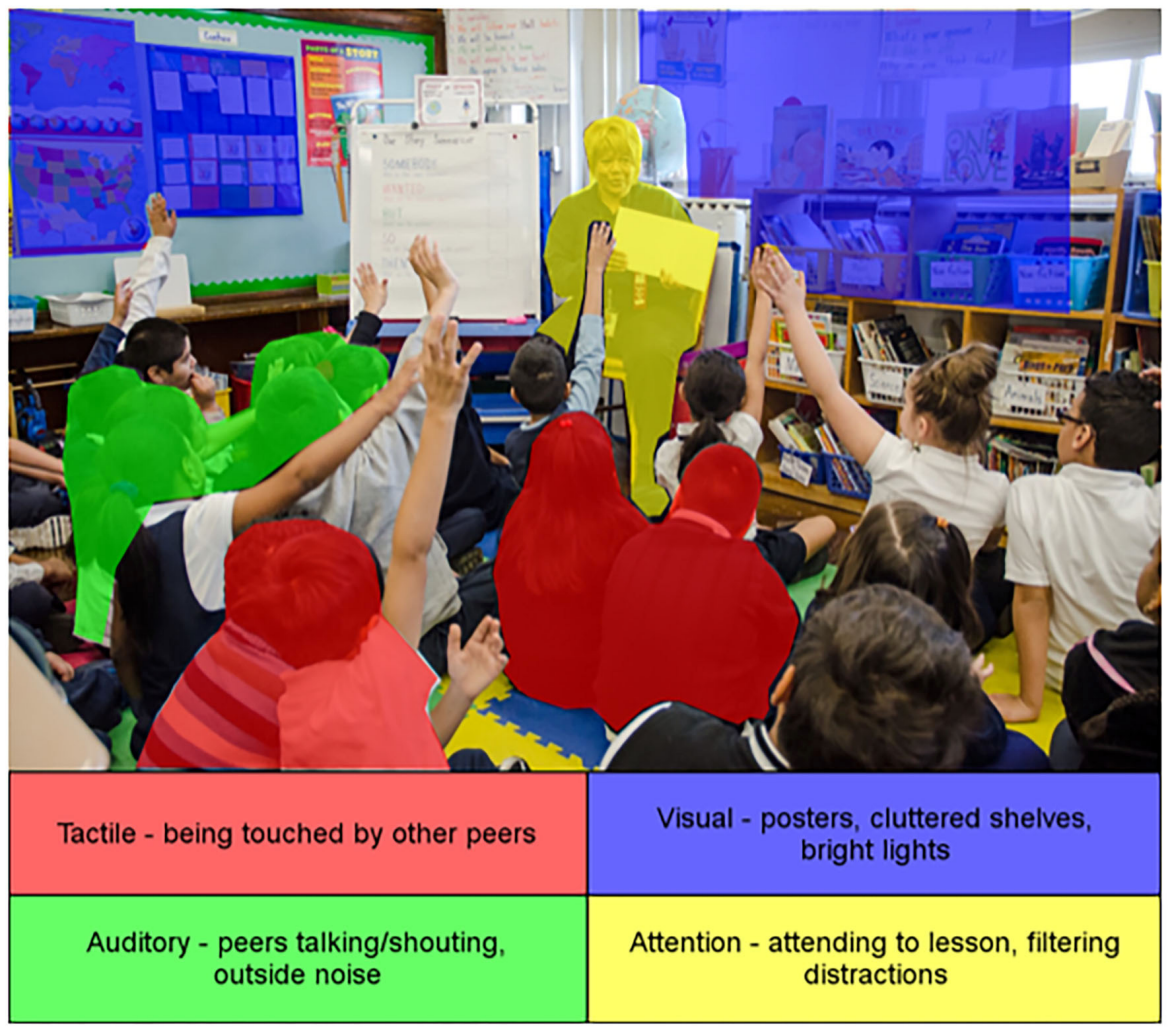
Example of the sensory and attentional demands that may be present in a classroom environment. Reproduced from https://catalog.archives.gov/.

Picture yourself in a classroom. The teacher is in the front of the class presenting new material. You—the student—are required to actively attend to this lesson while tuning out the shuffling of your neighbors' papers, the air blowing through a vent, and elaborate decorations on the walls (see Figure 1). Classrooms inherently challenge one's ability to process sensory information and focus attention. Although these demands change over time, they are present in all educational settings from preschool through postsecondary levels. Students must focus on a task or assignment in environments that contain distracting visual (e.g., other individuals moving, intense lighting), auditory (e.g., peer tapping their pencil, heating/air conditioning noise), and tactile (e.g., peers touching them in line) sensory input. In order to successfully navigate the classroom environment, students must appropriately react to sensory input and adaptively allocate attention to educationally-relevant information. Sensory processing of environmental stimuli has been shown to affect the participation of all students [e.g., (1)]. Likewise, attentional functions, such as filtering irrelevant classroom information and selectively attending to course-related agents, have been associated with academic achievement for typically developing (TD) students [e.g., (2)]. Therefore, it is critical to understand inter-individual differences in sensory processing and attention, and how students with sensory and attentional challenges function within classroom settings. One group of students that often exhibit differences in sensory processing and attention are students with autism spectrum disorder (ASD).

Autism spectrum disorder is a neurodevelopmental disability diagnosed on the basis of impairments in social interaction, verbal and non-verbal communication, and the presence of restricted and repetitive behaviors, including atypical sensory responsivity (3). Sensory and attentional differences have been widely studied in ASD. A large body of research has shown that school-aged children (4–7) as well as in adolescents and adults (8, 9) with ASD exhibit hyper- and/or hypo-reactivity to sensory input and atypical sensory interests [see (6, 10, 11) for more detailed reviews]. Additionally, although not included in the diagnostic criteria for ASD, a growing number of studies have also shown that individuals with ASD exhibit early and pervasive impairments in attention [see (12), for a review]. These sensory processing and attentional differences may present significant challenges to students in the classroom, negatively influencing access to educational material necessary for learning.

Since 2000, the prevalence of ASD has increased from 1 in 150 to 1 in 54 children (13) with a growing percentage of those diagnosed with ASD having average or above average intelligence (about 27–44%) (14). Additionally, earlier diagnosis and entry into evidenced-based early intensive behavioral intervention (EIBI) have resulted in improved outcomes for some preschool and school-aged children with ASD (15–17). The increase in prevalence and optimal progress associated with EIBI is reflected in greater enrollments, from preschool (18) through postsecondary education (19). As this trend continues, it is critical to ensure that the most efficacious accommodations and interventions are developed and that schools are providing the most appropriate, accessible environment for all students to meet their potential at every educational level.

The core and associated symptoms featured in ASD may, in part, help explain why students with ASD have demonstrated significant discrepancies between their intellectual ability and broad academic performance (20, 21). For example, Estes et al. (22) found that 60% of students with ASD performed below their predicted achievement level based on overall intellectual ability in at least one subject domain. Furthermore, at the postsecondary level, individuals with ASD are less likely to complete a degree than individuals with other disabilities (e.g., learning disabilities, speech/language impairment, hearing impairment, visual impairment, orthopedic impairments, other health impairment, and traumatic brain injury) (23). Together, these results suggest that students with ASD are not meeting their academic potential. Given the increasing number of students with ASD present in classrooms at all educational levels, understanding strengths and weaknesses in sensory processing and attention in ASD is necessary in order to provide optimal access. Therefore, the objectives of this paper are to: (1) provide a brief overview of the current literature on sensory processing and attention in individuals with ASD, (2) review how sensory and attentional functions may affect academic outcomes in both neurotypical and ASD learners, and (3) suggest potential accommodations or interventions for students with ASD based on these findings. As the goal for the current review was to integrate findings from disparate fields, a non-systematic approach was used.

## Sensory Processing

Sensory processing involves the perception, registration, and interpretation of different sensory stimuli in the environment. Dunn's Model of Sensory Processing (24) is commonly used to describe the inter-individual differences in sensory processing [e.g., (25, 26)]. Based on behavioral and neuroscience data, the model proposes that perception of sensory input is associated with one's sensory threshold, which is established as the pattern of interchange between habituation (when a stimulus is identified as familiar and the neural response is decreased) and sensitization (enhancement of neural transmissions to a stimulus) (24). In addition to sensory threshold, self-regulatory behavioral responses (passive, allowing sensory experiences to happen; or, active, using behaviors to dictate sensory experiences) also determine how sensory input is processed (27). An individual's sensory threshold and behavioral regulation determine the four patterns of sensory processing, including poor registration (high threshold/passive response, may be under-reactive/responsive, difficult to engage or withdrawn), sensitivity to stimuli (low threshold/passive response, may be hyper-active, over-responsive or distractible), sensation seeking (high threshold/active response, engaging in behaviors that increase their sensory experiences by adding movement, touch, sound, or visual stimuli), and sensation avoiding (low threshold/active response, may be resistant to participating in activities with unpredictable stimuli and prefer routines).

### Sensory Processing and ASD

The diagnostic criteria for ASD now includes atypical sensory responsivity, and several studies have described sensory dysfunction in this population using Dunn's model (9, 11, 25, 28–34). Summarizing the results of 14 prior studies, Ben-Sasson et al. (11) reported consistent sensory processing differences between ASD and TD groups, with the greatest differences in under-responsivity, over-responsivity and seeking categories across ages; however, not all individuals with ASD present with similar subtypes of sensory processing (5, 35). Extreme levels of sensory symptoms are present in ~95% children (ages 3–6 years old) (32) and adults with ASD (8), appearing to affect multiple sensory modalities (9, 31, 33), and are present across levels of ASD symptom severity (11).

Evidence of sensory processing differences also come from first-person accounts of both children (36) and adults (37–41) with ASD. Individuals with ASD reported that they seem to hear and attend to sounds that others do not notice, and had a difficult time ignoring these background noises, describing their experience as tiring and effortful (37). In order to cope with this, most individuals reported avoiding situations where they would anticipate issues, and some reported use of ear plugs to attenuate the sound (although with varying success) (37). Many adults with ASD describe their experience as “heightened senses” or more extreme than their TD peers (41). Additionally, when asked how individuals with ASD would design a building and what concerns they would consider, students with ASD consistently mentioned noise to be a major concern, indicating the importance of noise management in the classroom (38).

Finally, sensory dysfunction has also been associated with challenging behaviors that interfere with learning in the classroom as well as other social interactions. For example, Buyuktaskin et al. (27) found higher somatosensory temporal discrimination thresholds and an increase in self-, teacher-, and parent-reported behavioral or emotional problems. Many other studies have also found significant correlations between sensory difficulties and restricted, repetitive behaviors (RRBs) (42–44). More specifically, Chen et al. (42) found that the severity of sensory difficulties directly related to the frequency of RRBs reported. Furthermore, they showed that RRBs significantly impacted the completion speed on a short cognitive task. Other findings highlight the association between sensory processing differences and social communication impairments (45, 46). For example, Foss-Feig et al. (47) demonstrated that tactile seeking and hypo-responsivity are related to social impairments and non-verbal communication. Lastly, Marco et al. (48) suggest that language delays may be associated with differences in sensory processing. The relationship between sensory differences and verbal language and language acquisition has also been studied, demonstrating a relationship between sensory hypo-responsiveness and sensory seeking and poorer communication outcomes in the future (49, 50). Therefore, it is essential to provide accessible sensory environments in order to reduce the frequency of RRB behaviors, which may interrupt their learning and participation in the classroom and support social interactions between individuals with ASD and their peers.

## Attention

Broadly defined, attention can be thought of as collection of information processing operations that mediate the selection of information from simultaneous sources of internal or external input. This selection is controlled by top-down processes dependent on the expectations of the individual and bottom-up factors that are dependent on the physical characteristics of the stimuli. Accurate selection of relevant (and successful filtering of task-irrelevant) information is also dependent on the perceptual load of the stimuli (51). Petersen and Posner (52) and Posner and Petersen (53) outlined three attention networks: alerting, orienting, and executive control. The alerting network manages the state of increased sensitivity to stimuli and is divided into two components: tonic (general state of awareness/alertness that can be voluntarily maintained at a certain level, also known as sustained attention) and phasic alertness which is transitory and converts a state of rest to one that is responsive to other cues (e.g., behavioral cues) or novel information. The orienting network selects information from the incoming sensory information and involves disengaging, shifting, and reengaging the locus of attention. Lastly, the executive control network is made up of several different but integrated functions including inhibition, conflict resolution, planning, and cognitive flexibility.

### Attention and ASD

As discussed in previous reviews [see (12, 54) for example] individuals with ASD exhibit impairments in each attentional network. For the alerting network, individuals with ASD may demonstrate impairments of regulation of arousal and alertness levels (6), as well as differences in the phasic modulation of the alerting network (55); however, individuals with ASD evidence similar sustained attention compared to their TD peers [e.g., (56)]. As reviewed by others (57, 58) individuals with ASD exhibit consistent deficits in the orienting network across the lifespan, including impairments in disengaging and shifting attention to and from auditory and visual stimuli. Lastly, an uneven pattern of strengths and weaknesses in executive functions is present in ASD (59), with individuals with ASD often exhibiting relatively intact inhibition but impaired set shifting abilities. Additionally, research has consistently demonstrated that individuals with ASD have difficulties filtering irrelevant distractors. Specifically, when trying to maintain attention of a task-relevant stimulus, individuals with ASD show difficulty ignoring behaviorally-irrelevant distractors (60–64), including both visual and auditory information. For example, individuals with ASD have been shown to have an impaired ability to selectively attend to one sound source amongst several other competing sources (65), as a result of impaired filtering of irrelevant auditory information (62).

Some have hypothesized that the poor ability to filter out irrelevant information may be due, in part, to an increased perceptual capacity in individuals with ASD (60, 66, 67). Larger perceptual capacity may result in processing more of the information, including to-be-ignored input that is not relevant to the given task. Thus, an individual with ASD, who may have enhanced perceptual capacity, may be more likely to process task-irrelevant information and become distracted. While this increased capacity may result in certain advantages, for example enhanced visual search abilities (66), it may also contribute to greater distraction in low-load conditions. Together, the results from a growing body of research indicate that individuals with ASD exhibit early and pervasive impairments within each attentional network.

## The Role of Sensory Processing and Attention in Learning Environments

Classrooms provide a unique challenge for sensory processing. With the multiple visuals posted, numerous peers talking, noise from inside and outside the classroom, and close proximity of peers, students are exposed to multisensory stimulation from a variety of sources. Prior research by Dunn et al. (1) has shown that sensory experiences impact a child's participation whether or not they have any other neurodevelopmental condition, with a low threshold potentially resulting in increased negative behaviors and psychosocial states (e.g., anxiety, shyness), and a high threshold potentially leading to over-focusing on a stimulus, contributing to missing more cues in the environment (e.g., the teacher's instructions). Given the sensory processing differences associated with ASD described above, it is essential to evaluate and monitor the impact of complex sensory environments on engagement and academic achievement of students with ASD. In the following sections, the impact of auditory, visual, and tactile sensory processing on classroom participation for typically developing students and students with ASD is reviewed.

### Sensory Processing in the Classroom

#### Auditory Processing

##### Typically Developing Students

Learning in classrooms is heavily reliant on auditory information (e.g., from the teacher). In order to effectively access the educational material, relevant auditory content must be presented at a level that is louder than any irrelevant, background noise. Signal-to-noise ratio (SNR), the ratio of the strength of the target signal to that of the irrelevant, interference noise, has consistently been shown to be below recommended levels in classroom settings, suggesting that, at baseline, the classroom is likely to be a difficult listening environments (68). As the SNR becomes increasingly negative, greater listening effort is required, demanding more cognitive resources (69). Noise in the classroom has also been shown to have a detrimental effect on children's performance on letter, number, and word recognition as well as other academic test scores (70).

In addition, the type of auditory input is also associated with performance. Studies on the distractive effects of noise with TD individuals have shown that irrelevant social noise (i.e., speech) is more disruptive than non-speech noise on performance (71, 72). For example, Boets et al. (73) demonstrated that impaired auditory processing and speech-in-noise perception (along with categorical speech perception and phonological awareness) in kindergarten predict later difficulties with literacy in third grade students. Likewise, auditory processing, especially of sounds within the speech envelope (i.e., noise in the range of speech sounds that is similar to the fluctuations in fundamental frequency, amplitude, and duration of speech), has also been linked to early reading skills in TD children (74). Thus, poor SNR and the competing presence of irrelevant auditory stimuli may negatively affect the performance and participation of all students, especially if students have auditory sensory processing differences.

##### Students With ASD

A large body of previous research has shown that auditory stimuli to have the greatest negative impact on engagement and/or educational performance for students with ASD (4, 7, 75–78). Ashburner et al. (75) investigated the relationship between sensory processing and classroom emotional, behavioral, and educational outcomes. They found that sensory responses of children with ASD were significantly different from their TD peers. Of all the sensory modalities, differences in auditory processing appeared to most significantly impact daily functioning. Similarly, Howe and Stagg (76), based on a self-report questionnaire and a structured interview, identified hearing as having a significant impact on learning. Furthermore, Kanakri et al. (79) found a significant positive correlation between classroom noise levels and the frequency of repetitive behaviors. These results are consistent with teacher reports that noise control is crucial when working with students with ASD from preschool through high school (77). For example, Keith et al. (62) found that background noise added a significant stressor for individuals with ASD, with stress potentially leading to an increase in RRBs, further resulting in poorer academic outcomes. Lastly, in their review of SNR and its effect on the classroom performance of students with ASD, Van der Kruk et al. (80) reported that students with ASD benefited from improved signal-to-noise ratio as students demonstrated reduced ability to process speech in noisy environments. Therefore, atypical processing and sensitivity to environmental auditory stimuli present an immense barrier to accessing the educational curriculum for students with ASD.

#### Visual Processing

##### Typically Developing Students

The visual components, in particular the lighting and displays, of the classroom have been shown to impact student learning. Classroom lighting (specifically increased exposure to daylight from windows) has been observed to have a positive impact on student outcomes (81, 82). More recently, Barrett et al. (83) reported that the lighting in the classroom has a significant impact on all students' learning outcomes, stating that a combination of natural and electrical lighting is best. However, they cautioned against too much direct sunlight in classroom due to increased glare. The specific effects of different types of artificial (electrical) lighting have also been examined. A recent study compared student performance in LED and fluorescent light conditions, and showed that all students showed more engaged behaviors with the LED lighting condition, with the most change evident for students diagnosed with a developmental delay (84). Another study comparing the effects of LED vs. fluorescent lighting found that LED lights (blue-enriched lighting) can increase cognitive performance and alertness, as well as speed of cognitive processing and concentration, particularly in the morning (85).

The visual environment also impacts how students allocate their attention and process visually-presented information in the classroom environment. While elaborate, colorful visual displays (e.g., decorations, posters) are common in many classrooms, recent research has indicated potential negative effect on students' attention and performance in heavily decorated classrooms (86–88). This has been demonstrated in particular with kindergarten students, who were shown to be more distracted by the visual environment as seen by more time spent off task and smaller learning gains when the walls were highly decorated compared to when the decorations were removed (86). This negative impact of a highly-decorated visual environment has been shown to extend through the adolescent years (89). Furthermore, Tsubomi et al. (88) also suggest the need to reduce the load placed on students' visual processing by excluding as many visual distractions as possible to promote a successful learning environment in the classroom. Lastly, Boets et al. (90) demonstrated that visual processing has also been linked with orthographic ability, impacting later reading and writing development. Thus, it is critical to be cautious and aware of the different types of lighting and displays present in learning environments to ensure the most advantageous environment for all students to access the relevant classroom information.

##### Students With ASD

Many individuals with ASD report sensitivity to lighting (41), and demonstrate benefits in academic performance and participation with the use of less intense lighting (78, 91, 92). For example, Kinnealey et al. (78) showed improved attention and engagement for three adolescent students with ASD after installation of halogen instead of fluorescent lights. Additionally, teachers and occupational therapists who work with students with ASD described the beneficial effect of changing the lighting in the classroom on students' participation (91). As described previously, this aligns with other findings of classroom lighting impacting the engagement behaviors of all students, especially students with developmental delay (84).

Because individuals with ASD demonstrate difficulty with visual processing, it is important to consider the amount and content of what is being displayed in classrooms at all educational levels. Prior research suggests that classroom settings with a high amount of background visual displays were associated with poorer learning scores for all students following a mini-lesson, especially for students with ASD (93). These difficulties with visual attention have also been associated with poorer literacy and numeracy skills for children with ASD (94). However, limited and relevant visual displays may be helpful, as individuals with ASD demonstrate increased attention to the visually-presented background information (95). Considering the components of the visual environment in the classroom will allow for better accessibility to relevant classroom information while limiting exposure to distracting, unrelated material.

#### Tactile Processing

##### Typically Developing Students

To date, studies evaluating the effects of tactile processing in TD students in the classroom is limited, and, therefore, will not be reviewed.

##### Students With ASD

Although limited, clinical findings consistently describe tactile sensitivity in the classroom (48). Howe and Stagg (76) reported that touch/tactile was the second highest rated sensory modality that negatively affected students in the classroom. Likewise, Piller and Pfeiffer (91) also described the limiting nature of tactile stimuli (such as proximity to peers and interacting with different types of materials like cotton and glue) on the participation of preschoolers with ASD. A study focusing on school-aged children with ASD found that (along with auditory), touch was the most effected modality for the ASD group compared to TD peers in the classroom environment (4). This finding is supported by more recent research demonstrating that touch processing was highly impaired for students with ASD in the classroom (7). While understudied, the tactile domain clearly plays an instrumental role in the educational engagement of individuals with ASD as they engage with peers and educational materials in the classroom setting.

#### Attention in the Classroom for Typically Developing Students

In addition to sensory processing, a growing body of work has begun to show that academic skills and outcomes depend on more basic attentional functions. As outlined below, alerting, orienting, and executive control attentional networks may play a unique role in skills necessary for academic achievement. Carefully considering the role that each of these networks may have in educational engagement and success will elucidate the processes for learning and specific attentional areas that may be addressed to support the learning process.

Additionally, to investigate the long-term effect that attention may play in education, Rabiner and Coie (96) conducted a longitudinal study with over 300 students from kindergarten to fifth grade. The authors compared standardized attentional-problem measurements with reading achievement and found a strong correlation between attentional deficits and reading difficulties. Other studies support these findings and suggest a predictive relationship between attention and later academic achievement (97, 98).

Alerting function, which is associated with sensitivity and awareness of stimuli and the regulation of arousal and alertness levels, has been shown to be related to academic outcomes in students. Specifically, Razza et al. (99) demonstrated a predictive relationship between sustained attention and later academic outcomes for younger students. Furthermore, preschoolers' sustained attention has also been shown to impact students' engagement in middle school and indirectly effect adolescent math achievement (100). Steele et al. (2) investigated the predictive relationship between attentional abilities and academic achievement by measuring the performance of young students on reading, phonics, math concepts, and compared these with teacher-rated inattention and sustained and selective attention in students. Ultimately, they found strong associations between attentional functions and academic achievement, concluding that attentional processes are essential for the development of these skills. Prior research investigating the association between alerting functions and academic performance has also shown that in older students, greater school performance [measured by overall grades for the semester and grade point average (GPA)] is related to better sustained attention (101). Lastly, Stern and Shalev (102) found a significant relationship between accurate reading comprehension and longer duration of silent reading and sustained attention, concluding that good sustained attention is related to better reading comprehension as well.

Orienting attention—disengaging, shifting, and reengaging attention—to select relevant information is crucial for academic achievement. Erickson et al. (103) found that exogenously-driven selective attention was related to the performance of kindergarteners on classroom learning tasks. Additionally, Vogel et al. (104) conducted an fMRI study supporting a relationship between selective attention and the visual word form area (VWFA), a brain region that is associated with early reading skills and later literacy. Lastly, in a review by Stevens et al. (105), an influential link between selective attention and language processing and literacy was supported.

A large body of prior studies have shown a relationship between executive functions and academic achievement (106–118). For example, Tsubomi and Watanabe (88) demonstrated an association between visual working memory (or the ability to actively hold relevant visual information at a ready, accessible state) and visual inhibition of distracting, irrelevant information with elementary students' (age 7–12 years) academic performance. Findings from St Clair-Thompson (118) also indicate that executive functions (specifically inhibition and working memory) are associated with academic achievement and learning for school-aged children. Furthermore, difficulty in mathematical skills may be associated with poorer early executive function measures (set shifting, inhibitory control, and general executive behavior measures), suggesting a predictive relationship between executive function and later mathematical performance (110). This critical and predictive role of executive function is further demonstrated by a series of longitudinal studies (108, 119–121), a meta-analysis (111), and other focused research (107, 112), and suggest that executive abilities play a key role in academic outcomes.

### Attention in the Classroom for Individuals With ASD

Although there is extensive research on attention and academic achievement as described above, there are a limited number of studies focusing on the impact of attention in the classroom for individuals with ASD. However, given the reported pervasive impairment in attentional functions for individuals with ASD and the demonstrated importance of attention in academic performance for the TD population, it is plausible that attention impairments in individuals with ASD may exacerbate academic challenges. Additionally, as rated by educators, over half of their students with ASD were reported as under-achieving academically and demonstrated difficulty in maintaining attention in class (122). This variability in functional classroom performance may also be attributed to executive function deficits. Individuals with ASD have been shown to have uneven patterns of executive function (59), and, similar to their TD peers, potential deficits have been correlated with poorer overall school readiness measures, even in preschoolers (117). Additionally, the findings of May et al. (123) also suggest that poorer mathematical outcomes are linked to attentional switching difficulties. In a follow-up study, May et al. (124) showed that poorer reading performance was associated with difficulty with attentional switching. Individuals with ASD have also frequently demonstrated difficulty with written expression in school. Recent research has investigated the potential role of attentional deficits on this skill and have demonstrated a relationship between increased difficulty with attention and poorer written performance (125). Most recently, McDougal et al. (126) utilized the divided attention section of Test of Everyday Attention for Children (TEA-Ch) (127) to evaluate students' executive function in ways similar to how they would need to allocate attention in the classroom with school-aged students with and without ASD. They found a strong correlation between this divided attention measure and math achievement for all students, including students with ASD. Although research focused on the associations between attention difficulties and educational performance in ASD is limited, it is clear that attentional networks are essential for successful educational outcomes.

## Clinical Implications: Accommodations and Interventions

Together, the evidence reviewed above suggests that students with ASD may perceive academic environments as overwhelming and struggle with adaptively allocating attention in these settings. Both may contribute negatively to academic outcomes. Thus, designing classroom settings that address these barriers would allow for overall better access to the academic material. Although it is crucial to consider each student's (or group of students') sensory needs when deciding on environmental accommodations or interventions, there are many options that would be beneficial for all students no matter where they fall on the sensory response spectrum.

Because students with ASD exhibit unique sensory and attentional differences, analyzing their impact on classroom engagement, participation, and access and use of learning skills is essential. As discussed previously, effective attentional functions and sensory processing are crucial for successful academic outcomes as students navigate the classroom. However, when the classroom environment adds further demands on attention and sensory processing, students with ASD may particularly be at risk and demonstrate overall poorer performance and increased difficulty fully participating or engaging in the classroom activities. For example, research on neurotypical studies by Vogel and Schwabe (128) suggest that physiological responses to complex situations or environments (or stress) critically effects the process of memorization and learning new information. Therefore, identifying ways to decrease attentional and sensory demands and associated stress resulting from atypical sensory responses and/or the inability to filter out irrelevant sensory input by modifying the classroom environment is critical. Many studies have sought to address this concern and have described potential modifications to the classroom environment that would increase the accessibility of the educational material. Those recommendations are reviewed below.

### Sensory Accommodations

Environmental accommodations have the potential to affect all students in the classroom, as stated in the principle of Universal Design (UD), which is defined by Steinfeld and Maisel (129) as, “‘design for all' and represents an approach to design that incorporates products as well as building features which, to the greatest extent possible, can be used by everyone.” Universal design principles incorporate contextual modifications in order to meet the needs of a diverse range of individuals (130). Therefore, the use of many of these modifications would be beneficial for all students, including typically developing students and students with different disabilities.

### Auditory Accommodations

Many strategies have been suggested to target the auditory modality. In a review, Saggers and Ashburner (92) recently suggested the installation of sound absorbing walls, use of carpets, use of a “low-tech” sound meter or “high tech” noise level app to monitor the noise in the classroom, strategic use of the classroom space to reduce noise on communication situations, structured-turn taking and use of noise-reduction headphones for independent work. All of these modifications would allow for reduced competing irrelevant distractors as well as the overall noise level (improving the SNR). Other studies have investigated specific accommodations independently. Sound absorbing walls have been suggested as beneficial for improving attentional inhibition and auditory over-sensitivity as well (77, 78, 91). For example, Kinnealey et al. (78) found that implementation of sound absorbing walls led to an increase in students with ASD initiating social interactions with their peers. Sound-field amplification (SFA), a system that amplifies the teacher's voice above the ambient noise in the room for all students no matter where the teacher or students are in the classroom (131), has also been shown to benefit children with and without ASD by reducing auditory listening stress in the classroom by improving the SNR (80, 132, 133). FM systems, usually a personal system that transmits the sound source (teacher's voice) directly to the receiver (typically through the use of headphones), have also been studied as potential means to improve the SNR in the environment for students with ASD, although some students demonstrated difficulty with the tactile sensations of using the equipment/headphones that accompanied the systems (75, 134, 135). However, it should be noted that some studies indicate that predictable background noise may raise arousal levels and increase performance for those with attentional disorders (e.g., ADHD) and TD individuals (136, 137). For individuals with ASD, Keith et al. (62) demonstrated that predictable auditory stimulation can be beneficial for students in raising arousal for controlled or straight-forward tasks, allowing for a more comfortable environment for students who exhibit over-reactive or hyper-responsive sensory tendencies (44). Additionally, for inherently loud environments in a school such as the playground, hallways, and cafeteria, it is suggested that schools provide access to quieter spaces as well as lockers at the end of hallways for older students to limit exposure to these high-noise, stressful locations (92).

### Visual Accommodations

As previously described, many individuals with ASD prefer less intense lighting (41) and researchers have suggested this as a potential environmental modification that would be beneficial for students with ASD (78, 91). Hanley et al. (93) suggest limiting the visual displays put up in classrooms as well as careful consideration when creating the visual displays. However, Remington et al. (95) adds to this by emphasizing that including *relevant* visual information in the background display in the classroom can actually be beneficial for all students, not just students with ASD. Saggers and Ashburner (92) also suggest covering irrelevant classroom resources, using room dividers, placing desks so that they are not facing out the windows or other external distractors, and providing individual screens for students to use at their desk if needed. Therefore, inclusion of less intense lighting sources (e.g., halogen lights instead of fluorescent) and limited but relevant background visual displays would help provide a more accessible visual environment for students.

### Tactile Accommodations

Making spaces more predictable has been a coping strategy implemented by many adults with ASD (41) and has been suggested for classroom environments as well. For example, in order to provide a more manageable tactile environment, strategic spacing of students could reduce unpredictable tactile input and thereby reduce inattentive or distractible behavior in the classroom (75, 92). Weighted vests have also been suggested as potential accommodations in the past, however, more recent literature suggests that students with ASD experienced little to no benefit from these vests in the classroom (138–142). An additional commonly suggested tactile strategy includes implementation of flexible/alternative seating (e.g., sitting on therapy balls). The use of flexible seating allows for minimal movement and maintained arousal level to help students with ASD attend and engage in the classroom. Limited research has been done demonstrating efficacy of this strategy, however, results overall have shown a positive effect on classroom behavior and participation (143–145).

## Summary

Sensory accommodations are a critical step in creating optimal learning environments. Evaluating the noise levels, providing access to tools to enhance the target auditory stimuli (e.g., the teacher's voice), and providing quieter spaces for students will help reduce the burden on students' auditory processing and allow them to more easily focus on classroom salient information. Proper lighting and reduced clutter of visual displays in a classroom also provides a practical way to reduce strain and create easier access to relevant material. Lastly, while research on tactile accommodations is limited, implementation of flexible seating strategies may help maintain students' engagement in classroom activities. Utilization of these accommodations will ameliorate students' access and participation in the classroom setting.

Ultimately through implementation of modifications to reduce stress caused by sensory processing differences and to help address difficulty with inhibition and other attentional processes, students with ASD will be able to better participate in the classroom environment and engage in socially interactive opportunities in the classroom. This follows the principle of UD described above as these accommodations integrate contextual modifications into the classrooms that will address the needs of a diverse range of individuals (130). Therefore, the use of many of these classroom modifications would benefit a wide range of students with varying needs and would help create an optimal learning environment for all students.

### Attention-Targeted Interventions

As reviewed above, attentional functions play a key role in determining academic outcomes for all students. As such, attention-targeted interventions have been development in order to increase the efficiency of these abilities with the goal of improving academic outcomes. A review by Jacob and Parkinson (146) described several executive function interventions that have been shown to benefit TD students; concluding that computerized attentional training led to the most compelling improvement in attention. Spaniol et al. (147) demonstrated that an specific computerized attentional intervention, Computerized Progressive Attentional Training Program (CPAT) developed by Shalev et al. (148), led to improvements beyond attention including learning and general cognition for students with ASD. Additionally, Braingame Brian, another computerized executive function training that has been designed by Prins et al. (149) to improve working memory and attentional flexibility, has also demonstrated improvement in executive function, ASD-like behavior and quality of life; however, crucially this was not true for all children (particularly students who demonstrated increased difficulty with attentional flexibility) (150). The investigation of the effect of computerized games or tasks as attentional interventions is still preliminary in nature, and further studies should also consider the potential causal relationship between attention and academic achievement measures.

Other attentional interventions have been evaluated. For example, Kenworthy et al. (151) implemented the “Unstuck and On Target” (UOT) curriculum [developed by Cannon et al. (152) targeting executive functions, specifically those related to flexibility, planning, and goal-directed behavior] with third- through fifth-grade students with ASD and demonstrated greater post-intervention advances in problem-solving, flexibility, planning, and behavior in the classroom for students in this group compared to a control social skills intervention group.

Mindfulness programs (strategies aimed to teach purposeful shifting of attention and awareness to the present moments; training individuals to have control of their attention) have also been implemented to improve attentional functioning and executive function for individuals with ASD. Although this has been found to be effective in TD students (153), the effectiveness for individuals with ASD has had mixed reports. Juliano et al. (154) implemented an 8-week school-based mindfulness program with 24 students with ASD and found significant improvements in response inhibition and overall selective attention; however, Ridderinkhof et al. (155) failed to demonstrate a significant beneficial effect of the program, although showed trends suggesting improvement in orienting and executive function.

Hume et al. (156) also presented three interventions focused on shifting the responsibility of determining a response to environmental stimuli to the student to increase their independence (with an emphasis on decreasing adult prompting) including self-monitoring, video modeling and individual work systems. The self-monitoring intervention (where the student is required to pay attention to his/her own behavior, when they occur and their effects) has been shown to increase on-task behavior, thereby decreasing the negative effect of distractors. Video modeling (recorded performances of targeted skills that are repetitively watched to learn and eventually imitate the skill in real-world contexts) have been shown to increase adaptive behaviors and on-task behaviors. Additionally, implementation of predictable routines at all educational levels has also been shown to support executive functions by providing clear structure and help increase participation and social engagement in the classroom (157). Lastly, individual work systems [an element of the structured teaching system developed by Treatment and Education of Autistic and related Communication handicapped Children (TEACCH)] that focus on visually sequencing activities as incomplete or complete for students in a well-organized and defined work space with reduced distractions has also been shown to increase on-task behaviors and improve selective attention (specifically by modifying the environment to minimize extra visual and auditory information) (156, 158).

### Novel Targets for Intervention

Development of targeted interventions to address these attention and sensory difficulties have only recently been investigated methodically, and, thus, future research is necessary to replicate these results and determine their impact on academic outcomes. Novel accommodations and interventions addressing these difficulties based on empirical research of sensory and attentional differences in ASD could help address these potential adverse effects and enable students with ASD to reach their true academic potential. Thus, it is critical to continue to investigate and examine new and effective accommodation and interventions for individuals with ASD.

Considering the unique strengths of individuals with ASD may also provide insight on how to effectively design interventions. For example, a number of studies have now demonstrated that individuals with ASD evidence greater perceptual capacity citations. As such interventions and classroom modifications targeting inclusion of intentional, relevant classroom features (e.g., classroom posters, background music) may provide a novel direction for research. Remington et al. (95) provided preliminary evidence that the perceptual capacity of students with ASD can indeed be capitalized on when provided with task-relevant background material instead of distractors. Therefore, designing interventions that utilize the strengths of individuals with ASD in the classroom environment may provide a new realm of research for interventions, accommodations, and modifications for students that may also benefit all students.

Computerized interventions have continued to be developed and trialed. Most recently, Macoun et al. (159) investigated the effectiveness of a game-based cognitive training program using a “serious-game,” *Caribbean Quest* (CQ), to target attentional and executive function abilities in individuals with ASD. This study builds upon a previous study that had shown this particular game to improve divided attention, distractibility and working memory for a small sample (*n* = 7) of children with ASD (160). Using a hybrid cognitive approach (combing process-specific interventions with compensatory strategies), participants engaged with the gameplay in a one-on-one intervention session with a trained research assistant who taught metacognition strategies and supported generalization. CQ was broken into a mini-game structure specifically targeting inhibitory control, sustained and selective attention, and working memory, and the game progression was determined by participants' individual performance. Following 12 hours of training in this model, participants demonstrated preliminary improvements in academic measure (math fluency) and anecdotal reports from parents and teachers in attention, engagement, organization, flexibility, and working memory. This study provides preliminary support for the use of a “serious-game” model with interventionalist support to improve attentional and academic measures for children with ASD (159).

Additionally, recent research has started to investigate physiological responses of students with ASD in the classroom and other multisensory situations. For example, Corbett et al. (161) demonstrated that individuals with ASD exhibit increased physiological responses (elevated cortisol response) when playing as compared to their typically developing peers and an association of elevated responses with increased sensory sensitivity and parent-reported stress. Pfeiffer et al. (162) recently investigated sympathetic nervous system reactivity and auditory sensory sensitivities in students with ASD. They measured skin conductivity (electrodermal activity) of the participants in four phases of an auditory interventions. They found that sound hypersensitivity for individuals with ASD leads to those elevated sympathetic nervous system responses, which are then related to increased problem behaviors and stress. Through this study, they demonstrate that using wearable sensors in natural environments is an achievable way to gain information about the students' levels of arousal and stress. Therefore, it may be possible to utilize these tools to monitor and intervene when students with ASD are demonstrating difficulties with responding to their environment before problem behaviors, breakdowns, or exceeding their sensory threshold occur.

## Future Directions

Although research focused on the effects of the physical classroom environment on academic performance and outcomes has grown, there is still much to learn about how these may be linked in individuals with ASD. More specifically, further investigation of the impact of sensory and attentional deficits on the academic performance of students with ASD throughout the school years would provide a clear picture of how students with ASD are experiencing the classroom learning environment. Additionally, further systematic investigation of the impact of auditory, visual, and tactile sensitivity in the classroom for individuals with ASD is also crucial in order to provide sensory sensitive learning environments.

Several different sensory and attentional interventions for students with ASD have been studied, however, few have examined the interventions' impact on the students' academic performance. Further investigation of these interventions for students with ASD and their short- and long-term impact on academic performance would provide more guidance on how to address weaknesses in attentional functions and sensory processing in the educational setting. This research can then facilitate the creation of additional sensory- and attention-targeted environmental modifications and educational accommodations and interventions. Several empirical questions remain ripe for investigation:

- How are individuals with ASD impacted by complex, multi-sensory learning environments?
- How do comorbid conditions (such as attention-deficit/hyperactivity disorder; ADHD or specific learning disorders) impact classroom performance and academic outcomes?
- How can a more detailed understanding of the biological underpinnings of sensory and/or attentional differences in ASD enhance our ability to target these differences through interventions/accommodations?
- How can we capitalize on the attentional strengths of individuals with ASD (e.g., enhanced perceptual capacity)?
- What classroom modifications are beneficial in improving on-task behaviors, sustained attention, and distractor filtering for all students?
- How might pedagogical shifts (e.g., active learning environments) benefit or disadvantage students on the spectrum?

## Conclusion

Autism spectrum disorder is associated with atypical sensory responsivity and attentional impairments. These differences are present across the lifespan and have been shown to have a significant impact on the lives of students with ASD, from preschool through post-secondary education. Prior research has shown that classroom environments greatly impact engagement and academic performance for all students, including those diagnosed with ASD. Visual, auditory, and tactile stimuli in the classroom can be overwhelming, and filtering irrelevant sensory input is difficult in an unpredictable, multi-sensory environment. Modifications to the classroom environment may ameliorate sensory and attentional challenges faced by learners with ASD, and further benefit students with other disabilities as well as TD peers. Through design of an optimal sensory environment, students with ASD may more successfully access the educational curriculum. Likewise, through the implementation of attentional interventions, individuals with ASD may be given strategies and tools to help them fully engage in their academic environments, attend to essential educational material, and achieve their full academic potential.

## Author Contributions

CM and BK contributed to reviewing relevant literature and studies as well as writing sections of the manuscript. CM wrote the first draft of the manuscript. Both authors contributed to manuscript revision, read, and approved the submitted version.

## Conflict of Interest

The authors declare that the research was conducted in the absence of any commercial or financial relationships that could be construed as a potential conflict of interest.

## Publisher's Note

All claims expressed in this article are solely those of the authors and do not necessarily represent those of their affiliated organizations, or those of the publisher, the editors and the reviewers. Any product that may be evaluated in this article, or claim that may be made by its manufacturer, is not guaranteed or endorsed by the publisher.
